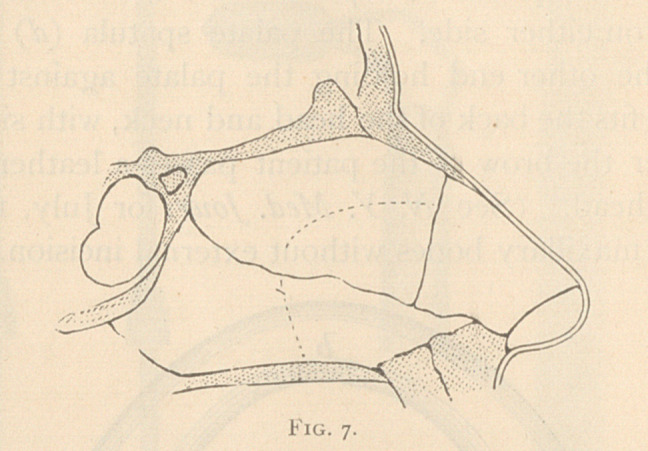# Extirpation of the Bones of the Nose and Mouth by the Use of the Surgical Engine

**Published:** 1880-01

**Authors:** D. H. Goodwillie

**Affiliations:** New York City; Late Clinical Assistant to the Metropolitan Throat Hospital; Permanent Member of the American Medical Association; Member of N. Y. Neurological Society; of the Medical Society of the County of N. Y.; of the Medical Library and Journal Association, etc., etc.


### EXTIRPATION OF THE BONES OF THE NOSE AND MOUTH BY THE USE OF THE SURGICAL ENGINE.

BY D. H. GOODWILLIE, M. D., D. D. S., NEW YORK CITY.

(Late Clinical Assistant to the Metropolitan Throat Hospital; Permanent Member of the American Medical Association; Member of N. Y. Neurological Society; of the Medical Society of the County of N. Y.; of the Medical Library and Journal Association, etc., etc.)

The deep-seated bones of the nasal fossæ become necrosed from numerous causes. The diagnosis is in many cases quite difficult, as in all cases there is stenosis of the nostrils from thickening of the soft tissue. But, by the aid of the rhinoscope under a very strong light, and by properly constructed nasal speculum and probe, together with the general physical condition and history in each case, it can be made out.

If necrosis is present to any great amount, it will generally be observed that the necrosed material will have made excoriated tracks on the pharynx on either side of the vertebral ridge.

Sometimes one, and again both sides, may be so seen. On the side on which the greater amount of disorganized tissue flows will be found the openings to the necrosed bone. If the vomer is the only bone necrosed, fistulous openings may be discovered—often near the junction of that bone with the palate posteriorly.

In many cases, owing to extensive swelling of the parts, rhinoscopy is impossible.

Necrosed bone cannot always be felt with a probe from the anterior nares, as the fistulous openings are in the posterior nares, and open toward the pharynx.

But, should there be necrosis of the soft parts, or if the bone necrosis extends to the maxillary bones, then it may be discovered by the probe.

The causes, in the writer's experience, have been in about the following order:

1. From a morbid virus in the system, in which syphilis stands most prominent, struma, diphtheria, etc.

2. From mechanical and traumatic causes; polypi, causing by their growth pressure and consequent necrosis; foreign substances, deviated nasal septum, blows upon the nose, etc.

In the nasal lesions in tertiary syphilis, the necrosis nearly always commences in the vomer, then extends to the other bones of the nasal fossæ. The first symptoms are an intense pain in the frontal sinuses, extending down the bridge of the nose, and, when the disease has extended to the hard palate, pain in the mouth, in the centre line of the palate.

*Treatment.*—When necrosis has been recognized, no time should be lost in removing it. The dissolution and discharge of a necrosed bone may cause the loss of the surrounding hard and soft parts. It will be necessary to give constitutional treatment suitable to each case. In December, 1872, the writer devised and made use of single and multiple revolving knives, saws, and trocars for operations upon the hard and soft tissues of the mouth and nose, the revolving power being supplied by the *surgical engine.* This consists of a fly-wheel, set in motion by the foot, a driving-pulley, and a communicating cord.

On the top of the upright movable shaft the pulley is connected to a flexible wire cable inclosed in a flexible sheath. This cable is connected to the hand-piece, in which can be put any revolving instruments. The flexibility of the wire cable allows the instrument in the hand-piece to be freely used at any angle. The hand-piece, held in hand as you hold a pen, is under perfect control. The instruments are securely fastened in the hand-piece by means of a spring catch.

FIG. 1.

The *single revolving knife* (Fig. 1) is circular and sharpened on the edge (*a*), and has a protecting sheath (*b*) to cover up the part of the knife left exposed.

Under a velocity of two or three thousand revolutions per minute, the single revolving knife, in cutting soft sensitive parts, gives little or no pain.

The *multiple revolving knives* (Fig. 2) are arranged around the end of a shaft in an acute angle, and *cut* as they revolve, and do not *scrape* as the dental burrs. These instruments have a protecting sheath (Fig. 3), to be used when necessary.

*Saws*, like the single knives, are circular, and have teeth on the edge.

Full size.

Fig. 2.

Fig. 3.—Knife (*a*) within the sheath.

The *trocars* are of different forms and sizes, and they are intended to make an opening and then to enlarge it. Fig. 4 shows two of the

Fig. 4.

most efficient ones: the spiral cutting edge, and the other flat, with two straight cutting edges and double edges on the point.

*Self-retaining nasal speculum* (Fig. 5) represented in the *Medical Record*, July 31st, 1875. The writer has several modifications of it to more effectually show the posterior nares.

*Oral speculum* described in the *Transactions of the State Medical*

Fig. 5.

*Society*, 1877, and consists of hard rubber or metal splint (*b*, *b*), covering upper and lower teeth, attached at their posterior ends by an adjustable hinge (*g*, *g*). The splints are separated and the mouth kept open by a brace (*a*, *a*) on either side. The palate spatula (*d*) is attached to lower splint, the other end holding the palate against the pharynx. The *head-rest* fits the back of the head and neck, with side pads; over these and over the brow of the patient passes a leather strap, firmly confining the head. (See *N. Y. Med. Jour.* for July, 1872, page 22. Resections of maxillary bones without external incision.)

Fig. 6.

*Operation.*—The patient is placed in an operating-chair, nitrous oxide is given to produce anæsthesia, and then ether is used, and the head securely fixed in the head-rest. If the operation is in the nose, now close off entirely the nasal pharyngeal opening by pushing the uvula and soft palate back against the pharynx by means of the palate spatula, which is attached to the oral speculum. This will prevent the necrosed portions of bone thrown from the revolving knives from entering the larynx. Where the operation is done without an anæsthetic, this preventive measure is not necessary. In the operation for the removal of the vomer, the knives are used to remove the anterior part of the necrosed bone (Fig. 7, shown by dotted line), and then the posterior part grasped with the forceps through the anterior nares.

Fig. 7.

This is pulled out from between the soft walls covering this part of the bone. When moved from its bed, it is either brought out through the anterior nares, or, if too large to pass there, it may be passed through the posterior nares. By the careful removal with the forceps of this posterior half of the vomer, the soft parts remain, and thus the union between the septum and palate is not destroyed. Have noticed in some cases a partial reproduction of that bone. In the case of the turbinated bones, they are entirely removed by means of the knives. The hard palate and maxillary bones can all be removed without disturbing in the least the soft parts. From a sinus big enough to admit the instrument can be successfully removed any amount of necrosed bone.

Before the patient recovers from the anæsthetic, and before the oral speculum is removed, the nasal cavity is syringed out with cold water, and cleared of all necrosed bone and blood. When sufficiently recovered from the anæsthetic, remove the speculum and unstrap the head. If the necrosed portions are too large to pass out of the anterior nares, let the speculum remain until the patient has regained consciousness, and then remove through the naso-pharyngeal passage or through any breach that may be made into the nasal cavity from the mouth.

The writer never makes use of sponges on cut surfaces in either mouth or nose, but makes use of antiseptic paper; or, if there is any hemorrhage from small vessels, it may be arrested by applying styptic paper. Have never yet had any secondary hemorrhage.

The writer has made use of the surgical engine for the successful removal of adhesions of the soft palate against the pharynx, nearly closing up the naso-pharyngeal passage. It has been successfully used in trephining the antrum, mastoid cells, exposing the superior and inferior dental nerves, opening abscesses, resections of the jaws, removal of epulic growths. Indeed, in many other surgical operations on any part of the body, it can be most efficiently used.

CASE I.—Mrs. C. T——, aged thirty-five years; born in New York; married November 25th, 1868. Up to this time quite healthy. Four months after marriage had syphilis, for which she received treatment by her family physician. Up to present time has had four births. Her first child still-born at six months; second child born at full term and lived a week; third child still-born at eighth month; fourth child born at full term and lived ten months.

In 1872, had syphilitic laryngitis and was salivated. She came under my care in November, 1874. On examination I found necrosis of the vomer, lower portion of the ethmoid, vault of the hard palate, and inferior turbinated bones of both sides, and alveolus of the intermaxillary bone. There was a hole in the hard palate a half inch in length. Front teeth quite loose from necrosis of the maxillary bone. These were at once removed. Rhinoscopic examination very difficult to make, as the uvula and soft palate were much swollen. Large ulcers on the pharynx. To combat the specific poison the patient was put upon iodide of potassium, two grammes, and increased to four grammes a day, with tonics and nourishing food.

April 29th, 1875, operated for the extirpation of the necrosed bones. There were present, Drs. A. C. Post, J. T. Darby, Leonard Weber, L. B. Bangs. All the necrosed bones were removed by the revolving multiple knives through the opening in the palate and through the nostrils. The necrosed palatal vault, both inferior turbinated bones, and a small portion of the vomer, were removed through the opening in the palate; through the nostrils, all the necrosed portion of the

3

maxillary bone and the anterior portion of the vomer and ethmoid. The posterior portion of the vomer was now seized with the forceps and removed. By this means the soft parts covering the vomer were left intact, so that by a rhinoscopic examination the posterior part of the septum was seen as before the operation. In this case there appeared to be a reproduction of bone in this part of the vomer, and to some extent of the hard palate.

A few days after, removed by the knives some small necrosed portions of the intermaxillary, after which the parts healed rapidly. The voice somewhat nasal in tone until the opening in the palate was closed.

In October, 1875, about six months after the extirpation of the necrosed bones, uranoplasty was performed for the closure of the opening in the hard palate, which was now three-fourths of an inch in length. After removing the mucous membrane from edges, an incision is made on each side of the fissure through the soft parts and newly formed bone of the hard palate.

The soft parts were cut through by means of a galvano-cautery knife, and so had no bleeding. The bone is now pierced by the drill, and the bone separated by a chisel after the method of Sir William Ferguson; or it may be sawed through, and then they are sprung together and the fissure thus closed. In this case four horse-hair sutures were used to hold the flaps together.

These side-incisions must be kept open by packing them, or removing the granulations each day, to prevent healing until the edges of the fissure are united. A gutta-percha splint is now fitted and worn over the palate. This prevents the food, fluids, and air from causing disturbance to the healing process.

I present wax models of this case taken from casts of it before, during, and after completion of the operation.

It will be seen that the external appearance of the nose has not altered in shape, notwithstanding the nasal septum and bony palate, upon which it rests, are gone. Have never seen the nose fall in except when the cartilage or nasal or maxillary bones were involved—in other words, the bridge of the nose.

CASE II.—Mrs. F. C——, aged twenty-one years, born in New York State, was sent to me by Dr. J. Marion Sims. She was married in 1865; then quite healthy; has had three still-born children, and one now living.

In January, 1872, had inflammation of the brain, which was afterward followed by inflammation of the bowels. In 1873 had severe neuralgic pains on the bridge of the nose, centre of the hard palate, and left side of the face. This was followed by a swelling in the centre of the hard palate, and all the upper teeth were extracted. In December, 1873, when she came under my care, her condition was as follows: Her physical powers were very much reduced; constant pains in her head; a hole in the left canine fossa; great discharge from the nose and mouth. By rhinoscopic examination, and by a probe through the hole in the canine fossa, I discovered necrosis of the nasal septum and turbinated bones of both sides.

The specific origin of disease being recognized, she was put upon iodide of potassium, tonics, cod-liver oil with phosphates. December 26th, as there was a good deal of pain and swelling of the nasal septum, it was lanced, and bled freely and gave her great relief. January 4th, 1876, lanced the nasal septum again. February 3d, periostitis of the left nasal bone externally appeared; applied a leech. February 4th, swelling and pain gone. February 9th, patient having improved in strength, but still suffering intense pain, removed all the necrosed bone by the revolving knives. In this operation removed the vomer, lower portion of the ethmoid, inferior and middle turbinated, maxillary walls of both right and left antrum, and a good portion of the palate. Present, Drs. George A. Peters, E. L. Keyes, F. R Sturgis, and G. H. Fox. February 10th, found the patient going about the house attending to some of her household duties; no pain since the operation. February 13th, removed small pieces of the intermaxillary bone. March 6th, had some swelling of the left side of the nose, extending under the eye.

Feeling herself so much better after the operation, she had neglected to take the potassium as ordered, and this is the penalty of such disobedience. Ordered a leech and increased the dose of the iodide of potassium to four grammes per day. March 8th, swelling very much reduced and pain nearly gone. March 10th, pain and swelling gone. There was a small amount of pus on the left side of the nose, which was drawn away with the aspirator. April 10th, patient expresses herself as being nearly well. Iodide of potassium reduced to two grammes every other day. Cod-liver oil to be continued. June 23d, '76, patient now quite well, and by a rhinoscopic examination no discharge was discovered. There now only remains the opening of the canine fossa to be closed.

CASE III.—*Necrosis of Turbinated Bones from Scrofula.*—Miss E. J. A——, aged twenty years, has had discharges for some time. Smelling much impaired. On examination discovered that both middle turbinated bones were necrosed. Considerable bulging of the nasal septum to the left side, which, her mother says, came from a fall in childhood. Removed the necrosed turbinated bones with the revolving knives, while she was under anæsthesia produced by nitrous oxide. After a month's treatment the parts healed, respiration free through the nostrils, and she was discharged.

CASE IV.—The following case was brought to me by Dr. Leonard Weber, of this city: William H——, of New York, aged thirty-two years, with syphilitic necrosis of the bones of the nasal fossæ. His condition was found as follows: Small hole through the hard palate one-half inch in length; four fistulous openings—above the alveolus, at the left central incisor, on each side of the left canine, and above the first molar of the same side. Some teeth were extracted on this side; the sound and firm teeth were allowed to remain.

In the presence of Drs. L. Weber, C. C. Lee, R. P. Lincoln, T. R. Pooley, H. G. Fox, L. Spannhake, and E. C. Lining, U. S. A., there were removed through the opening in the palate, and through the nostrils, the hard palate, vomer, inferior turbinated bones, cancellated portion of the left maxillary and intermaxillary bones.

The posterior portion of the vomer was dislodged and removed by the forceps, without separating its covering from the palate. In these extirpations there has never been any great amount of bleeding, and have never yet had to resort to the tampon. The styptic action of the paper controls all bleeding from the small vessels. There was much thickening of the soft parts, just inside of the vestibule of the nostrils; and as it interferes with free respiration, it was removed by means of the galvano-cautery. A protecting shield is put into the nostril, the top part of which incloses the part to be cauterized. The white-hot cautery wire is applied through the shield to the part exposed at the top of it.

CASE V.—H. W. B——, from Otsego county, New York, had catarrhal difficulty when a child. Has had polypi removed from right nostril by family physician. In July, 1876, the writer removed a large polypus from right nostril, attached by a large pedicle to upper part of vomer. From pressure the left middle turbinated bone had been lost, and from the same cause the vomer was pushed to the left. The right inferior turbinated was forced down into the inferior meatus.

There were three bends in septum. The greatest bend was in the posterior and upper part of the septum ; the lesser bend in the cartilaginous septum. The whole septum had also a very sharp bend, with hypertrophy of the bone along the line of, and bending into, the inferior meatus. This, with a pushing downward by the growth of the inferior right turbinated, produced complete stenosis of that nostril. This warping of the septum into the inferior meatus probably commenced with his trouble in childhood. This condition of things prevented the free discharge of mucus from the nostrils. In September, '76, removed with the nasal punch a portion of the bend in the cartilaginous septum. When I saw him again in May, '77, the upper part of the vomer had necrosed and passed away, the lower thick hypertrophied part was removed under an anæsthetic. The multiple knife, armed with a shield, passed through the inferior meatus, cutting its way through to the pharynx. The inferior turbinated bone was also removed. This gave a clear passage for the escape of the mucus and free respiration.

The most common local application used in these cases is a powder consisting of iodoform and camphor, each four grammes, subnitrate of bismuth, thirty-two grammes, blown into the nostrils with several pounds' pressure, so as to reach every part of the nasal cavity. To do this efficiently the powder must be impalpable, the calibre of the blower small, and applied with considerable force of air, the parts to be thoroughly cleansed with salt and tepid water by means of a syringe, and then the powder applied through the anterior nares.—*Medical Record, N. Y.*

## Figures and Tables

**Fig. 1. f1:**
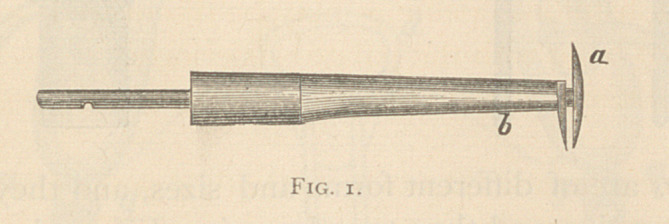


**Fig. 2. f2:**
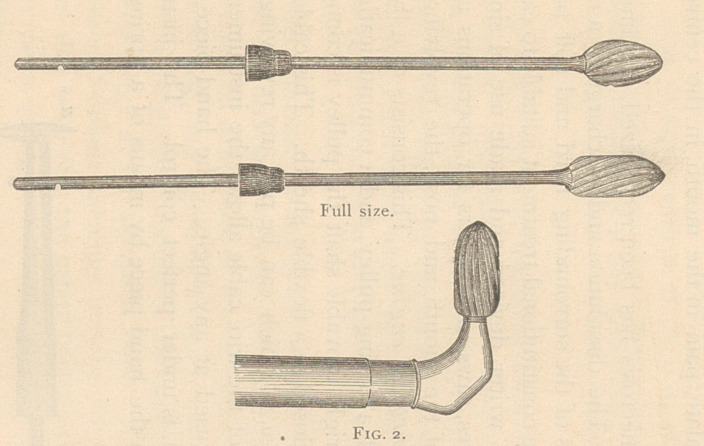


**Fig. 3. f3:**
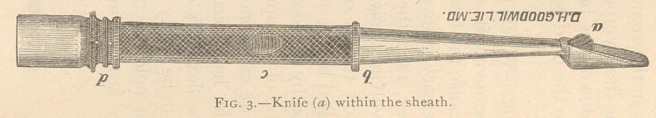


**Fig. 4. f4:**
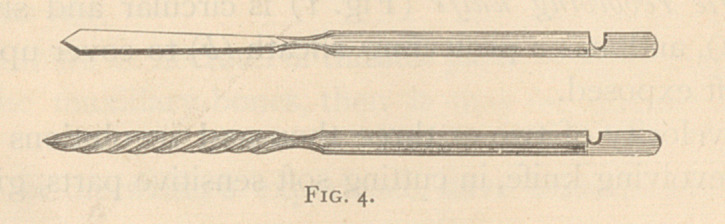


**Fig. 5. f5:**
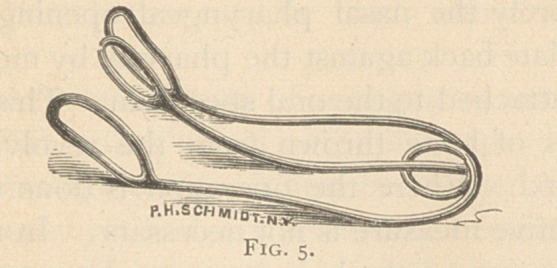


**Fig. 6. f6:**
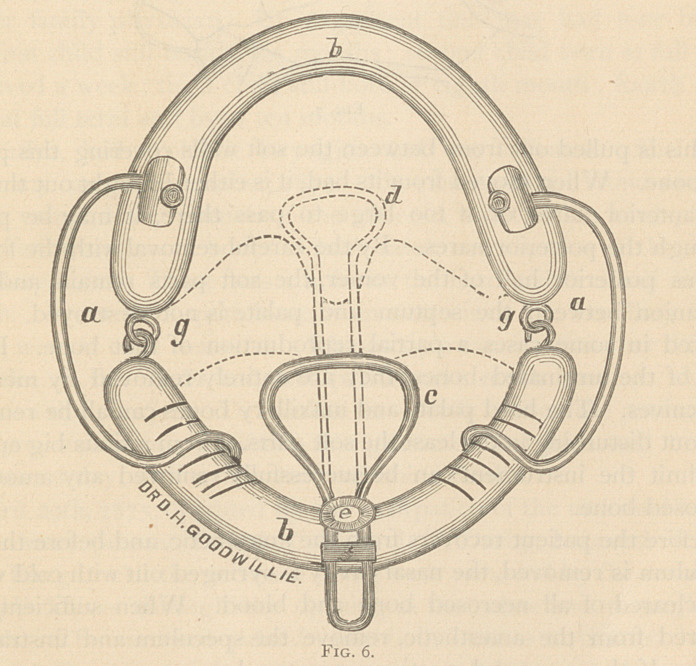


**Fig. 7. f7:**